# Oncogenic driver mutations in lung cancer

**DOI:** 10.1186/2213-0802-1-6

**Published:** 2013-03-08

**Authors:** Susan Y Luo, David CL Lam

**Affiliations:** Department of Medicine, University of Hong Kong, 102 Pokfulam Road, Hong Kong, SAR China

**Keywords:** Lung cancer, Driver mutations, EGFR, ALK

## Abstract

Lung cancer is a heterogeneous and complex disease. Genomic and transcriptomic profiling of lung cancer not only further our knowledge about cancer initiation and progression, but could also provide guidance on treatment decisions. The fact that targeted treatment is most successful in a subset of tumors indicates the need for better classification of clinically related molecular tumor phenotypes based on better understanding of the mutations in relevant genes, especially in those oncogenic driver mutations. *EGFR* gene mutations, *KRAS* gene mutations, *EML4*-*ALK* rearrangements and altered *MET* signaling are widely recognized alterations that play important roles in both the biological mechanisms and the clinical sensitivity to treatment in lung cancer. In this article, we reviewed the discovery of the clinical values of these oncogenic driver mutations and the clinical studies revealing the prognostic and predictive values of these biomarkers for clinical sensitivity and resistance to anti-EGFR therapy or other targeted therapies. These form the basis of personalized treatment in lung cancer based on biomarker profiles of individual tumor, leading to therapeutic advancement and betterment.

**Electronic supplementary material:**

The online version of this article (doi:10.1186/2213-0802-1-6) contains supplementary material, which is available to authorized users.

## Review

Lung cancer is the leading cause of cancer-related death worldwide. In the past, therapeutic decisions have been based on histological classifications, which distinguish small cell lung cancers (SCLC) and non-small cell lung cancer (NSCLC). The latter comprises three major subtypes: squamous cell carcinoma, large cell carcinoma, and adenocarcinoma [1]. Although the histological features have been proven to play an important role in the selection of chemotherapy [1], the overall survival remains very poor as a result of presentation of disease at advanced stage.

A diversity of genomic and epigenetic abnormalities has been reported in NSCLC. Oncogenic driver mutations refer to mutations that are responsible for both the initiation and maintenance of the cancer. These mutations are often found in genes that encode for signaling proteins that are critical for maintaining normal cellular proliferation and survival. The presence of mutations on these genes will confer growth advantage on cancer cells, favoring their being selected during tumor progression [2]. NSCLC, especially lung adenocarcinomas, can be further sub-classified by their genetic mutation profiles, making personalized treatment strategies based on the identification of oncogenic driver mutations feasible.

Epidermal Growth Factor Receptor (*EGFR*) gene mutations were the first targets for targeted treatment in NSCLC. Clinical efficacy and outcomes of EGFR-tyrosine kinase inhibitors (EGFR-TKI) have been reviewed thoroughly [3–7]. Deletions in exon 19 and the missense mutation L858R or L861Q in exon 21 exhibit an association with favorable response to reversible EGFR-TKIs whereas the secondary mutation T790M in exon 20 and insertions in exon 20 will confer resistance to gefitinib and erlotinib, while the nature and clinical sensitivity of other less common mutations like those mutations in exon 18 in the tyrosine kinase (TK) domain of *EGFR* are not well defined [8].

Apart from *EGFR* targeted therapy, more molecular targeted agents have developed to improve therapeutic outcomes, for example, bevacizumab which is an angiogenesis inhibitor targeting vascular endothelial growth factor (VEGF) [9], pemetrexed inhibiting thymidylate synthase and other folate dependent enzymes [10], as well as a mammalian target of rapamycin (mTOR) inhibitor everolimas [11]. Meanwhile, other oncogenic mutations that can define clinically relevant molecular subsets of NSCLC have been identified. The presence of individual driver gene is usually found to be mutually exclusive to each other. In this article, we review the four commonly known oncogenic driver mutations in lung cancer – *EGFR* mutations at exons 18 – 21, *KRAS* gene mutation at codons 12 and 13, *EML4-ALK* fusion genes and deregulation of *MET* signaling.

### *EGFR* mutations

The epidermal growth factor receptor family of tyrosine kinases consists of four forms: *EGFR* (*ErbB1*, *HER1*), *ErbB2* (*HER2*), *ErbB3* (*HER3*) and *ErbB4* (*HER4*) [6]. The binding of specific ligands leads to homo- and hetero-dimerization, with subsequent autophosphorylation of the intracellular receptor TK domain. The activated TK activity subsequently recruits appropriate downstream components of the TK signaling pathway which are involved in multiple cellular processes, including cell proliferation, cell survival, cell motility and cell invasion [12].

**Figure 1:**
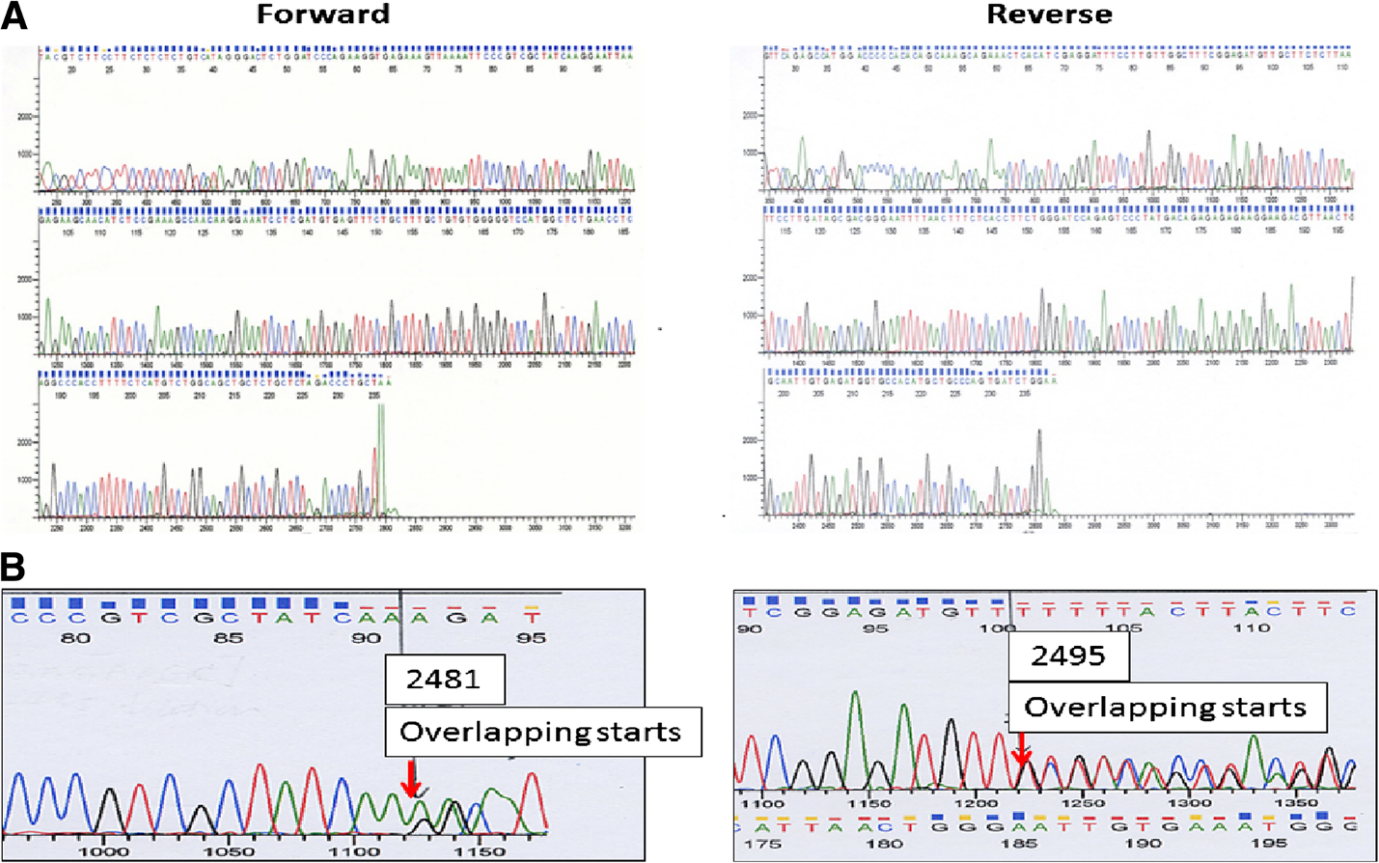
**Analysis of** ***EGFR*** **exon 19 mutation by direct sequencing.** (**A**) Wild-type *EGFR* exon 19; (**B**) An example of inframe deletion in *EGFR* exon 19 (2481_2495del). The arrows indicate the span of deletion in each amplified sequence of exon 19.

**Figure 2:**
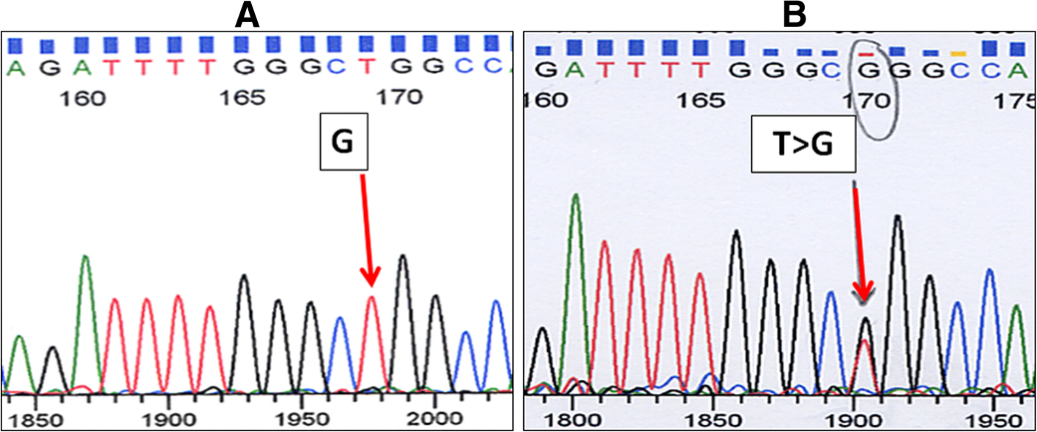
***EGFR*** **exon 21 mutation analysis via direct sequencing.** (**A**) Wild-type *EGFR* exon 21; (**B**) One missense mutation (L858R) in *EGFR* exon 21. The arrow indicates the position of the mutant nucleotide. Only forward sequences are shown.

*EGFR* mutations have been extensively studies in NSCLC with about 27% overall incidence (according to COSMIC database, http://www.sanger.ac.uk). *EGFR*-mutant NSCLC, which often exhibits adenocarcinoma histology, has been found to be associated with a better prognosis compared to *EGFR* wild-typed NSCLC in most populations [13–18], except for a study in Chinese patients [19]. The activating *EGFR* mutations have been identified in exons 18 to 21 of the TK domain, about 90% of which are deletions in exon 19 (Figure 1) and the point mutation L858R in exon 21 (Figure 2) [6]. The mutant *EGFR* shows a preferential binding of gefitinib or erlotinib to ATP, thus correlating with higher sensitivity to these two anti-*EGFR* TKIs [20, 21]. Several trials have revealed the clinical role of activating *EGFR* mutations in EGFR-TKI therapy. A randomized prospective Phase III study (NEJ002) with 230 Japanese advanced, untreated and *EGFR*-mutant NSCLC patients sustained improved progression-free survival in the first-line gefitinib versus standard chemotherapy [22]. According to a study in Spain, erlotinib also showed similar effectiveness in *EGFR*-mutant patients [23].

Some *EGFR* mutations, although they occur in exons 18 to 21, are related to primary resistance to EGFR-TKIs, for example, small insertions or duplications in exon 20. Additionally, the presence of other types of mutations can induce acquired resistance to *EGFR* inhibitors, such as T790M (exon 20), L747S (exon 19), D761Y (exon 19) and T854A (exon 21) [24]. Other genetic alterations contribute to primary or acquired resistance to EGFR-TKIs, including *KARS* mutations, *PIK3CA* mutations, loss of *PTEN* function, *MET* amplification and altered *EGFR*-related signaling [25]. Therefore, strategies to overcome resistance to EGFR-TKIs have been under investigation, including development of second- and third-generation of EGFR-TKIs, combinational therapy targeting compensatory pathways, alternative TKI dosing to delay drug resistance, or continuation therapy with EGFR-TKIs [13, 25].

Apart from erlotinib and gefitinib, second-generation irreversible EGFR-TKIs have been under extensive investigation, for example, afatinib and dacomitinib. Afatinib (BIBW 2992; Boehringer Ingelheim; Ingelheim, Germany) is a highly selective and irreversible inhibitor of both *EGFR* and *HER2* [26]. It has been found to be effective in NSCLC with *EGFR* mutations, especially with de novo T790M mutations [27]. Several phase III trials of afatinib are undergoing, including LUX-Lung 3), in which afatinib is compared to cisplatin/pemetrexed in the first-line treatment of advanced NSCLC patients with activating *EGFR* mutations, as well as LUX-Lung 6 comparing afatinib with cisplatin/gemcitabine in the same population as LUX-Lung 3 with more recruitment in China, India and South Korea. Dacomitinib (PF-00299804, Pfizer; New London, CT, USA) is an irreversible pan-HER inhibitor, which showed remarkable activity to gefitinib-resistant *EGFR* T790M mutations or *HER2* mutations [28]. A phase I/II study of this inhibitor in Asian populations who were refractory to chemotherapy and erlotinib or gefitinib, 15% response rate and 32% 6-month overall survival achieved [29]. Therefore, a randomized phase III trial (JBR-26) comparing dacomitinib to placebo in the third-line treatment in patients have failed chemotherapy and EGFR TKIs is ongoing.

### *KRAS* mutations

A milestone study in 1984 identified a *KRAS* mutation in a squamous cell lung carcinoma with the absence of this point mutation in the corresponding normal bronchial or parenchymal tissue [30]. Since then *KRAS* mutations have been found frequently in NSCLC, and according to clinical trials, the incidence of *KRAS* mutations in NSCLC ranges from 8% to 24% [31]. Most activating *KRAS* mutations in NSCLC are located in codons 12 or 13, and are also reported in lung adenocarcinomas [32]. The study in which *KRAS* mutations were analyzed in nearly 500 lung adenocarcinomas showed that *KRAS* mutations were found in 15%, 22% and 25% of tumors from never smokers, former smokers and current smokers respectively [33]. Whereas *EGFR* mutations tend to occur more frequently in never-smokers with lung cancer, the presence of *KRAS* mutations cannot be easily predicted based on smoking status alone [34]. Another novel finding of this study was that *KRAS* transition mutations were more common in never smokers, whereas former/current smokers were more likely to harbor *KRAS* transversion mutations. This implies that transversions may be smoking related.

The mutated *KRAS* proteins exhibit impaired GTPase activity, resulting in constitutive activation of *RAS* signaling. Since *KRAS* is downstream of *EGFR*, constitutive activation of *KRAS* renders resistance to anti-EGFR therapy. Many studies have observed lower efficacy of EGFR-TKI therapy in *KRAS* mutated NSCLC patients [35, 36]. The presence of *EGFR* mutations and *KRAS* mutations are mutually exclusive in the same tumor. However, there was a report on a case series of three patients with both activating *EGFR* mutations and *KRAS* mutations demonstrated that all EGFR^+^/KRAS^+^ patients showed a positive response to gefitinib or erlotinib [37]. Several large clinical observational studies failed to identify *KRAS* mutation to be a significant response predictor to EGFR-TKIs [38–40]. As a result, *KRAS* positivity cannot be established as a criterion to exclude NSCLC patients from EGFR-TKI therapy.

The development of therapeutic agents targeting mutated *KRAS* signaling is under intensive investigation. An obstacle is that mutant *KRAS* proteins entail loss-of-function. It is more difficult to inhibit loss-of-function proteins than gain-of-function ones like mutant *EGFR*. Current approaches try either to inhibit protein synthesis of mutated *KRAS* or to impede downstream effectors of mutant *KRAS*.

### *EML4-ALK* rearrangement

The anaplastic lymphoma kinase (*ALK*) protein is a receptor tyrosine kinase in the insulin receptor superfamily. In 2007, Soda *et al.* identified a small inversion within the short arm of chromosome 2, resulting in the fusion of the N-terminal of the echinoderm microtubule-associated protein-like 4 (*EML4*) gene with the *ALK* gene [41]. Up to now, at least 11 different *EML4-ALK* variants have been reported, all of which involve the coiled-coil domain of *EML4* and the intracellular tyrosine kinase domain of *ALK* [42]. Furthermore, all *EML4-ALK* variants exhibit dimerization and constitutive activation of the fusion proteins [42, 43].

There is no “gold standard” method to screen the *ALK* gene rearrangements. Three detection technologies have been evaluated, including fluorescence *in situ* hybridization (FISH), reverse transcriptase PCR, and immunohistochemistry (IHC). The frequency of *EML4-ALK* fusions in unselected NSCLC patients, according to previous studies mostly with East Asians, ranges from 1.6% to 11.6% [42, 44–46]. Although other histological sub-types rarely contain *EML4-ALK* rearrangements, lung adenocarcinoma has been reported to be the major type showing *EML4-ALK* translocations. The majority of Caucasian lung adenocarcinoma harboring *EML4-ALK* show the signet-ring cell histology, whereas the acinar pattern is pre-dominant in *ALK*-positive Asian adenocarcinomas [47]. *ALK*-positivity tends to be found in younger NSCLC patients [44]. Similar to *EGFR* mutations, *EML4-ALK* fusions are associated with never or light smokers [48]. *EML4-ALK* translocations and *EGFR* mutations are mutually exclusive except for rare cases [45].

In Shaw *et al.* study, wild-type NSCLC patients and *ALK*-positive NSCLC patients displayed a similar response to chemotherapy and no significant differences in overall survival, however, *EML4-ALK* translocations have been found to be associated with resistance to EGFR-TKIs [44]. Similarly, *EML4-ALK* fusion gene was not a significant prognostic factor based on the analysis of 720 resected lung adenocarcinomas [49]. Consistent with these findings, *EML4-ALK* status did not affect the sensitivity of advanced NSCLC patients to platinum-based combination chemotherapy in terms of response rate and progression-free survival although overall survival of *EML4-ALK* positive patients tended to be shorter than that of the *EGFR* mutated cohort nut resembled that in the wild-type cohort [50]. In contrast, according to a study including *EGFR* wild-type and advanced-stage lung adenocarcinoma patients who received either monotherapy or platinum-doublet chemotherapy, *ALK* positivity conferred superior overall survival [51]. Compared with aforementioned studies, this study enrolled more *EML4-ALK* translocated patients (39 out of 116, 34%), which may partially explain better outcome. Additionally, in Chinese patients with advanced NSCLC, response rate to EGFR-TKI was similar between *ALK* rearranged and *EGFR* mutated patients although median progression-free survival was significantly shorter in those with *EML4-ALK* gene [52]. The apparent discrepancy between findings of this study and that of the study by Shaw *et al.* may be explained by predominantly Asian population in this study and the limited sample sizes in both studies. The clinicopathological features of *EML4-ALK* rearrangement in NSCLC patients needs further investigation.

In order to treat *ALK*-positive patients, selective tyrosine kinase inhibitors of *ALK* are currently under clinical trials, including an *ALK/MET* inhibitor PF-02341066 (crizotinib) [53]. Unfortunately, acquired resistance will emerge in *EML4-ALK*-positive tumors. Several secondary mutations in the *ALK* gene, such as L1196M and C1156Y, have been revealed to contribute to resistance to crizotinib [54, 55]. In order to overcome resistance, new molecules continue to be developed. CH5424802, a selective, potent, and orally available *ALK* inhibitor, have exhibited remarkable activity against C1156Y- and L1196M-resistant *EML4-ALK* mutants [56]. Clinical trials have been conducted to confirm safety and efficacy of this agent in *ALK-* positive NSCLC [46].

### *MET* signaling

The *MET* gene, which is located on chromosome 7, encodes a receptor tyrosine kinase composed of an extracellular α-chain and a transmembrane β-chain. The natural ligand for *MET* receptor is hepatocyte growth factor (HGF), also called scatter factor (SF). Since *MET* interacts with numerous downstream effectors, the activation of *MET* signaling affects many pathways and regulates various cellular processes, including cell proliferation, cell motility, cell scattering, cell invasion, metastasis, angiogenesis and epithelial to mesenchymal transition (EMT) [57–59]. Therefore, it is not surprising that deregulation of this signaling pathway can be considered as a driving force in tumor initiation and tumor maintenance.

In transformed cells, *MET* can be altered via overexpression, genomic amplification, mutations, or alternative splicing [57, 60, 61]. These alterations result in aberrant *MET* activation which can be mediated through HGF-dependent or HGF-independent mechanisms.

*MET* overexpression has been observed in NSCLC. In a study using 32 lung cancer tissues, all tumor samples expressed *MET* with no significant *MET* staining in corresponding normal lung tissues, and 61% (14 of 23) of NSCLC showed strong *MET* expression examined by IHC. Furthermore, an increase in *MET* activity identified by higher levels of phosphorylated *MET* (p-*MET)* at sites Y1003 and Y1230/1234/1235 came along with *MET* overexpression in this study. It was also mentionable that the activated p-*MET* was preferentially expressed in tumor cells located at the invasive front of NSCLC tissues [62]. In another study with 183 lung adenocarcinomas, *MET* amplification was observed in 8 (4%) patients with wild-typed *EGFR* and wild-typed *KRAS*, indicating that the presence of *MET* gene amplification might be mutually exclusive with *EGFR* and *KRAS* mutations. Phosphorylation of *MET* at sites Y1234/1235 has been found to be associated with poor survival in patients who have complete resection of lung tumors [63].

*MET* gene copy number variations have also been reported in NSCLC. *MET* status was analyzed with FISH in 447 NSCLC patients and high *MET* gene copy number (≥5 copies/cell) was observed in 48 cases (11.1%), and patients with high *MET* gene copy number (*MET*-positive) exhibited shorter survival than *MET*-negative patients [64].

Mutations provide another mechanism for *MET* dysregulation. Mutated *MET* allows the kinase to overcome inhibitory mechanisms, thus becoming constructively activated or hyperresponsive to stimuli. Mutations could also prolong the duration of stimulating signals by increasing the level of activation or by preventing the degradation of the kinase. One study examined individual exons of semaphorin, juxtamembrane, and tyrosine kinase domains of *MET* in 141 East Asians, 76 Caucasians and 66 African Americans [65]. The results showed that *MET* mutations varied with ethnicity. N375S, occurred within the semaphorin domain, was the most frequent non-synonymous mutation, and the frequency of this mutation was higher in East Asians compared to Caucasians. In both East Asians and Caucasians, the frequency of N375S was higher in squamous cell carcinoma than in other non-small cell lung cancer. Among East Asians, the frequency of N375S in males was much higher compared with females [65]. Since there is a relationship between ethnic differences and *MET* mutations, greater knowledge of this correlation can help us understand incidence, prognosis and treatment of lung cancer.

Several agents targeting *MET* signaling are under investigation. Among them, a non-ATP competitive MET inhibitor, tivatinib (ARQ197), has been used in combination with erlotinib (EGFR-TKI) as second-line treatment for previously-treated non-small cell lung cancer [66]. The dual MET-EGFR combinatorial inhibition is well-tolerated in advanced stage lung cancer patients. Although no significant change in progression-free survival (PFS) or overall survival (OS) has been reported in the intent-to-treat population, improvement in PFS and/or OS can be seen when including key prognostic factors and/or biomarkers, for example, presence of *KRAS* mutations. More *MET*-targeted agents are under preclinical and clinical studies, making *MET* the next major biomarkers in lung cancer.

### Other oncogenic driver mutations

The identification of oncogenic driver mutations reveals the complexity and heterogeneity of NSCLC. A collaborative study investigated 623 candidate cancer genes in 188 lung adenocarcinomas. 26 genes were discovered to be somatically mutated at high frequencies and thus may be related to tumorigenesis, including *ERBB3*, *ERBB4*, *VEGFR*, multiple ephrin receptors genes and *NTRK* genes [67].

### Significance of oncogenic driver mutations in lung cancer severity and therapy

**Figure 3:**
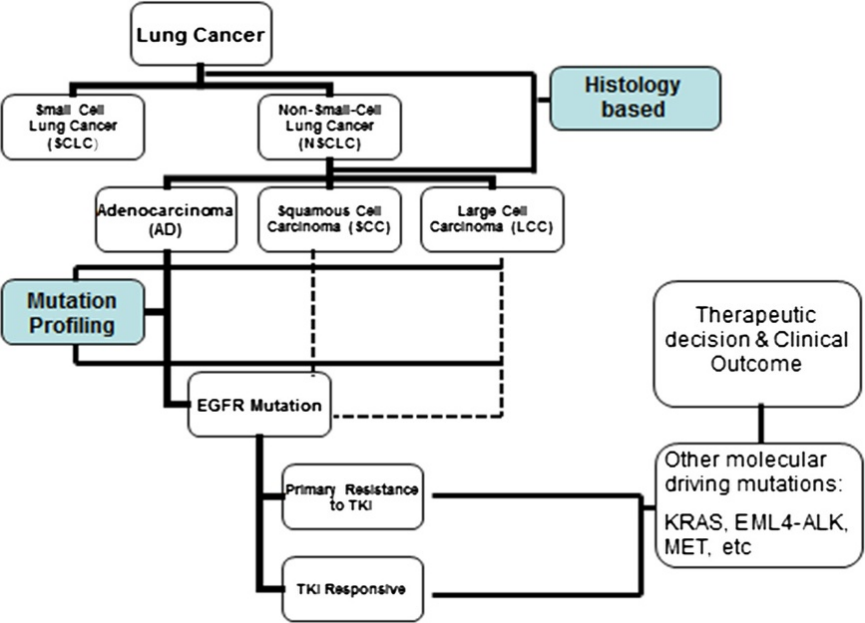
**A suggested schema for guidance of clinical testing for oncogenic driver mutations which aid in personalized treatment in lung cancer.**

Table 1 lists these four molecular targets with their respective detection methods and inhibitors. The presence of oncogenic driver mutations leads to a phenomenon called ‘oncogene addiction’ wherein tumor cells tend to be dependent on the specific mutant oncogene for their own survival and growth. Blocking the relevant oncogenic pathway by specific inhibitors may induce ‘oncogenic shock’ which ultimately results in cancer cell apoptosis [68]. This hypothesis suggests that the promising future of lung cancer treatment is indeed personalized therapy with drugs targeting specific driver oncogenes that “drive” tumorigenesis. The selection of proper therapeutic approach should be based on both histological features and the tumor mutation profiles of individual patient (Figure 3). This combination can contribute to better prediction of the malignant behavior and to improved clinical management. *EGFR* mutations could be present in early stage NSCLC [69, 70], suggesting that it may be possible to detect of lung cancer at more early stages via the molecular testing of mentioned driver mutations in susceptible individuals.

**Table 1:** **Summary of common oncogenic driver mutations, their corresponding testing methods and their respective inhibitors**

Target	Detection method	Inhibitor
**EGFR**	Direct sequencing	Gefitinib, Erlotinib
	Real-time PCR	BIBW2992 (Afatinib)
	Single-strand conformational polymorphism	PF00299804 (Dacomitinib)
	High-resolution melting amplicon analysis	HKI-272 (Neratinib)
		BPI-2009 (Icotinib)
		EKB-569 (Pelitinib)
		CI-1033 (Canertinib)
		GW572016 (Lapatinib)
**KRAS**	Direct sequencing	Not available
	Real-time PCR	
	Amplification refractory mutation system (ARMS)	
	Restriction fragment length polymorphism (RFLP)	
	Co-amplification at lower denaturation temperature-polymerase chain reaction (COLD-PCR)	
**ALK Fusion**	Fluorescence *in situ* hybridization (FISH)	PF-02341066 (Crizotinib)
	Immunohistochemistry (IHC)	CH5424802 (AF802)
	Real-time Reverse Transcription-PCR	
**MET**	Quantitative PCR	PF-02341066 (Crizotinib)
	Fluorescence *in situ* hybridization (FISH)	ARQ197 (Tivantinib)
	PCR-based sequencing	GSK1363089 (Foretinib)
		XL184 (Cabozantinib)
		PF-04217903
		SGX523

## Conclusion

As we further our understanding into the biological mechanisms underlying these oncogenic driver mutations, the clinical relevance of these driver mutations will allow for further advancement into targeted therapeutics in lung cancer.

## Authors’ original submitted files for images

Below are the links to the authors’ original submitted files for images. Authors’ original file for figure 1
Authors’ original file for figure 2
Authors’ original file for figure 3
