# Supplementary figures and images for: Oncogenic driver mutations in lung cancer
Source: Transl Respir Med. 2013 Mar 8;1:6. doi: 10.1186/2213-0802-1-6 (PMC6733434; doi:10.1186/2213-0802-1-6)

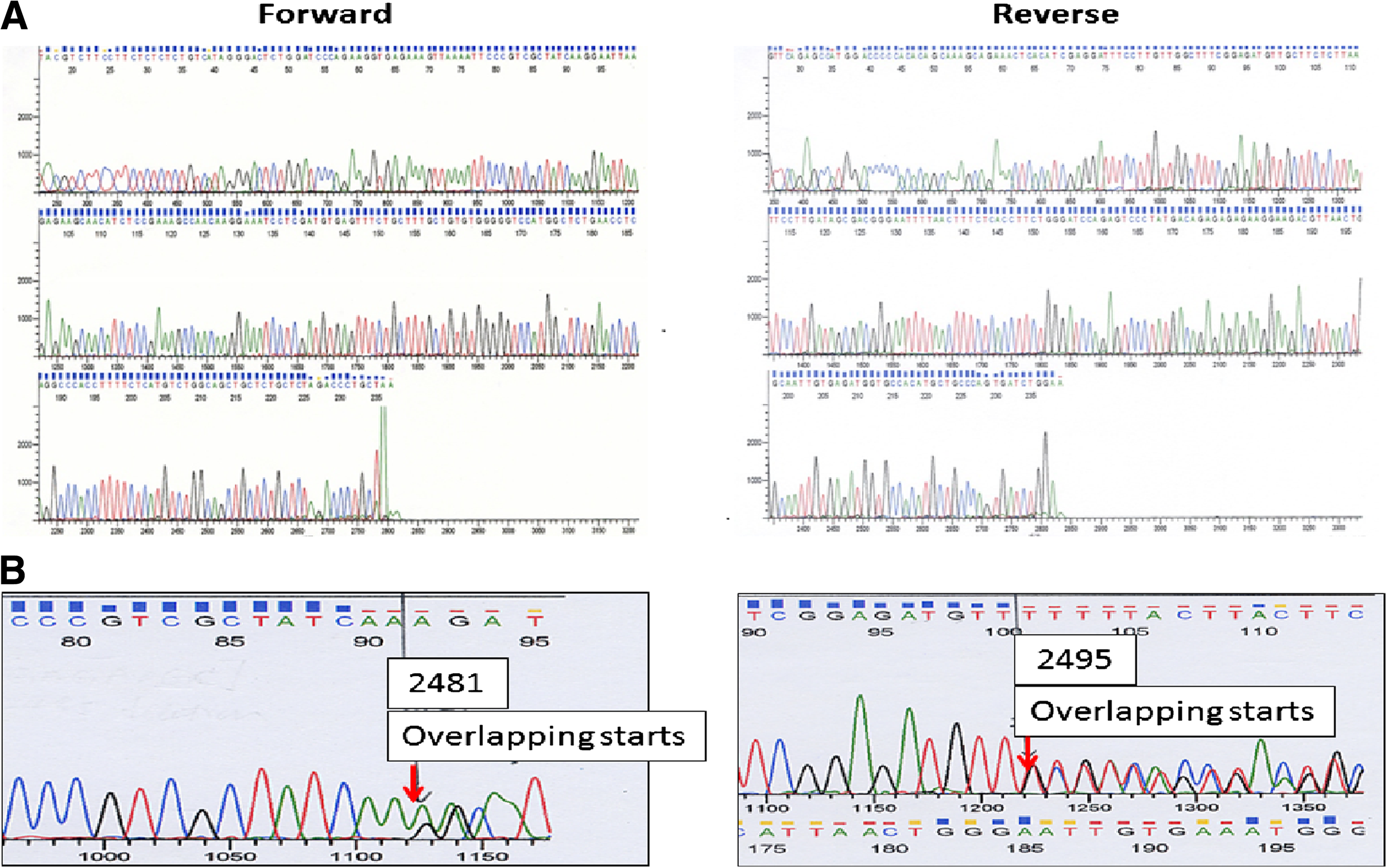

Supplement: Supplementary file 1 — Authors’ original file for figure 1 [file 40247_2012_7_MOESM1_ESM.tiff]

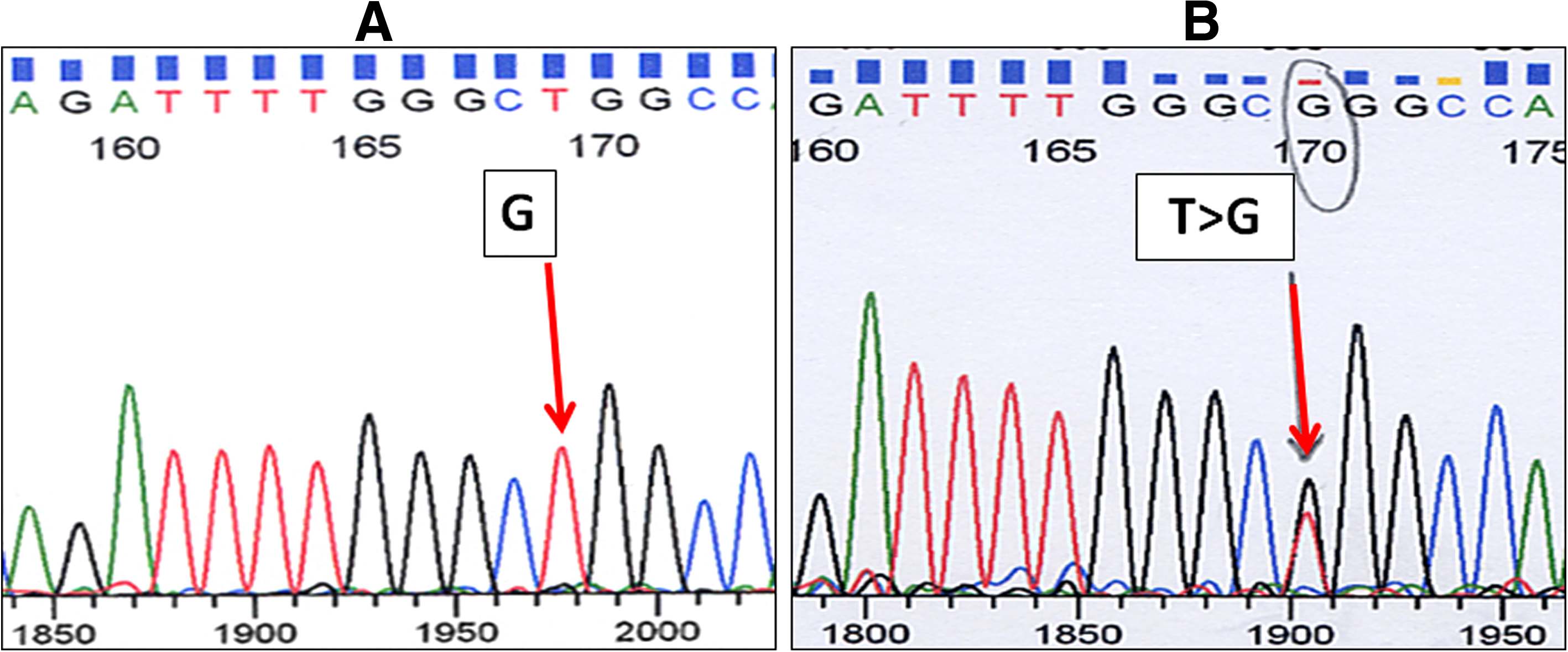

Supplement: Supplementary file 2 — Authors’ original file for figure 2 [file 40247_2012_7_MOESM2_ESM.tiff]

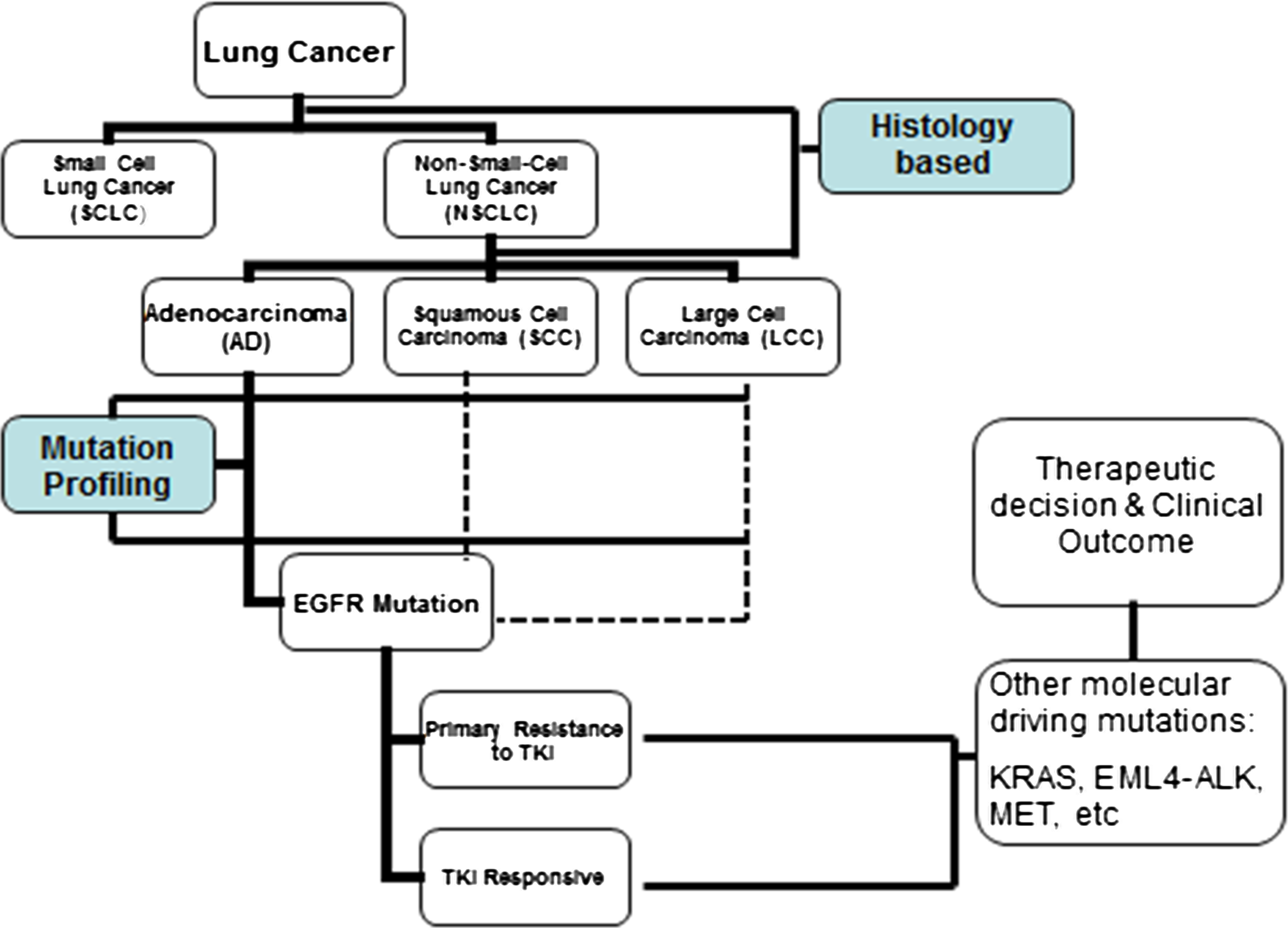

Supplement: Supplementary file 3 — Authors’ original file for figure 3 [file 40247_2012_7_MOESM3_ESM.tiff]
